# Meso-scale reorganization emerges despite broad convergence in plant–bee interaction networks across the honeybee's native and non-native ranges

**DOI:** 10.1098/rsbl.2026.0436

**Published:** 2026-11-11

**Authors:** Leandro Hachuy-Filho, Caio S. Ballarin, João Vitor de Alcantara Viana, Pedro Augusto Lacerda-Barbosa, Felipe W. Amorim

**Affiliations:** ^1^ Laboratório de Ecologia da Polinização e Interações (LEPI), Departamento de Biodiversidade e Bioestatística, Instituto de Biociências, Universidade Estadual Paulista ‘Júlio de Mesquita Filho’ (UNESP), Botucatu, São Paulo, Brazil; ^2^ Programa de Pós-graduação em Zoologia, Instituto de Biociências, Universidade Estadual Paulista ‘Júlio de Mesquita Filho’ (UNESP), Botucatu, São Paulo, Brazil; ^3^ Programa de Pós-graduação em Biologia Vegetal, Instituto de Biociências, Universidade Estadual Paulista ‘Júlio de Mesquita Filho’ (UNESP), Botucatu, São Paulo, Brazil; ^4^ Department of Animal Biology, University of Campinas (UNICAMP), Campinas, São Paulo 13083-862, Brazil

**Keywords:** *Apis mellifera*, plant–bee interaction, biological invasions, ecological homogenization, network convergence, plant networks, pollination network resilience

## Abstract

The western honeybee (*Apis mellifera*) has been introduced far beyond its native range, potentially reshaping plant–pollinator interactions. However, whether its integration into ecological networks differs between native and non-native regions remains poorly understood. We conducted a systematic review and compiled 80 quantitative plant–bee interaction networks encompassing the native and non-native ranges of *A. mellifera* to test whether such differences emerge consistently across three organizational levels: overall network architecture (macro-scale), participation in motif classes (meso-scale) and species-level structural roles (micro-scale). Macro- and micro-scale metrics did not differ between ranges, indicating broad convergence in network architecture and species-level structural roles across the honeybee's global distribution. However, motif composition differed consistently between ranges, with *A. mellifera* participating more frequently in fan motifs and less frequently in core–peripheral motifs in non-native regions. These findings suggest that, outside the native range, honeybees are more often embedded in asymmetric interaction structures that may reflect a more dominant pattern of resource use. Overall, our results reveal that while invasive generalists can promote structural convergence in mutualistic networks at both macro- and micro-scales, consistent network reorganization emerges at the motif level, suggesting that honeybee introductions may influence how floral resources are shared among pollinator species.

## 1. Introduction

Pollinator communities and the ecosystem services they provide are increasingly shaped by anthropogenic pressures [1,2]. One of the most pervasive outcomes of the Anthropocene is the large-scale movement of species across biogeographic boundaries through human activities [3]. Introduced species can alter community composition, disrupt species interactions and modify ecosystem functioning, including processes fundamental to angiosperm reproduction, such as pollination [4,5]. Because pollination underpins both plant reproduction and community stability [6], understanding how introduced pollinators integrate into native interaction networks is key to predicting their long-term ecological consequences.

Among introduced pollinators, the western honeybee (*Apis mellifera*) is exceptional for its ecological breadth and global reach. Native to Africa, Europe and parts of western Asia [7,8], honeybees have been introduced on all continents except Antarctica for apiculture and crop pollination [3,9]. Traits such as eusociality, generalist foraging and nesting flexibility facilitate its establishment across a wide range of ecological contexts [10–12]. Local-scale studies have shown that *A. mellifera* can outcompete native bees through exploitative competition for nectar and pollen, alter foraging behaviour and visitation frequencies and act as a reservoir of pathogens affecting wild pollinators [13,14]. These shifts may negatively affect native bee populations by reducing resource availability and altering abundance or fitness, while also affecting plant pollination through changes in visitation patterns and pollen deposition, potentially reducing reproductive success when honeybees are less effective pollinators than native species [14–16]. However, effects are context-dependent, with stronger impacts often reported in natural or insular systems [4,17,18], whereas in intensively managed agricultural landscapes, honeybees may partially compensate for pollinator loss (e.g. [17,19–21]). Such effects may also differ between native and non-native ranges. Within its native range, *A. mellifera* shares a longer ecological and evolutionary history with local plant and pollinator communities, whereas in non-native regions it interacts with communities without a shared evolutionary history. This contrast, together with differences in ecological context and management, may influence how honeybees integrate into local interaction networks and provide a basis for expecting their structural roles to differ between ranges.

Such species-level shifts can cascade through entire mutualistic networks, affecting community-wide interaction patterns. By interacting with many plant species, *A. mellifera* may increase connectance (i.e. the proportion of potential plant–pollinator interactions that actually occur) and nestedness (i.e. a pattern in which interactions are organized around a core of highly connected species shared by more specialized species), while reducing modularity (i.e. the extent to which species form distinct groups of interacting partners), thereby enhancing apparent functional redundancy but decreasing overall network specialization [21–23]. Importantly, macro- (the overall organization of species interactions), meso- (network motifs, i.e. small recurring interaction patterns that form the building blocks of ecological networks) and micro-scales (the roles of individual species) capture complementary aspects of network organization and may therefore reveal different signatures of structural change. These scale-dependent effects may remain undetected when only macro- or micro-scale properties, the most commonly used levels of network analysis, are considered [24], potentially leading to an underestimation of invasion impacts.

Ecological networks can be characterized across complementary organizational levels, but these levels differ substantially in how frequently they have been investigated and in the structural information they capture. While macro-scale metrics summarize whole-network architecture and micro-scale metrics describe species-level roles, the meso-scale, represented by network motifs, captures recurring patterns of direct and indirect interactions among small groups of species and remains comparatively understudied [25,26]. Notably, networks with similar macro-scale properties can differ markedly in their meso-scale organization, suggesting that ecological responses obscured by whole-network descriptors may emerge at this intermediate level [25,26].

Here, we performed a systematic review of the literature and compiled 80 quantitative plant–bee interaction networks spanning the native and non-native ranges of *A. mellifera* (figure 1). We asked whether differences in honeybee integration between ranges are consistently expressed across levels of network organization or emerge only at particular scales. To address this question, we quantified network properties at three complementary levels: (i) macro-scale (overall network architecture), (ii) meso-scale (participation in distinct motif classes), and (iii) micro-scale (species-level structural roles). By integrating these levels, we evaluate whether broad structural similarity between ranges may conceal finer reorganization in how *A. mellifera* participates in local interaction patterns across the globe [24].

**Figure 1.**
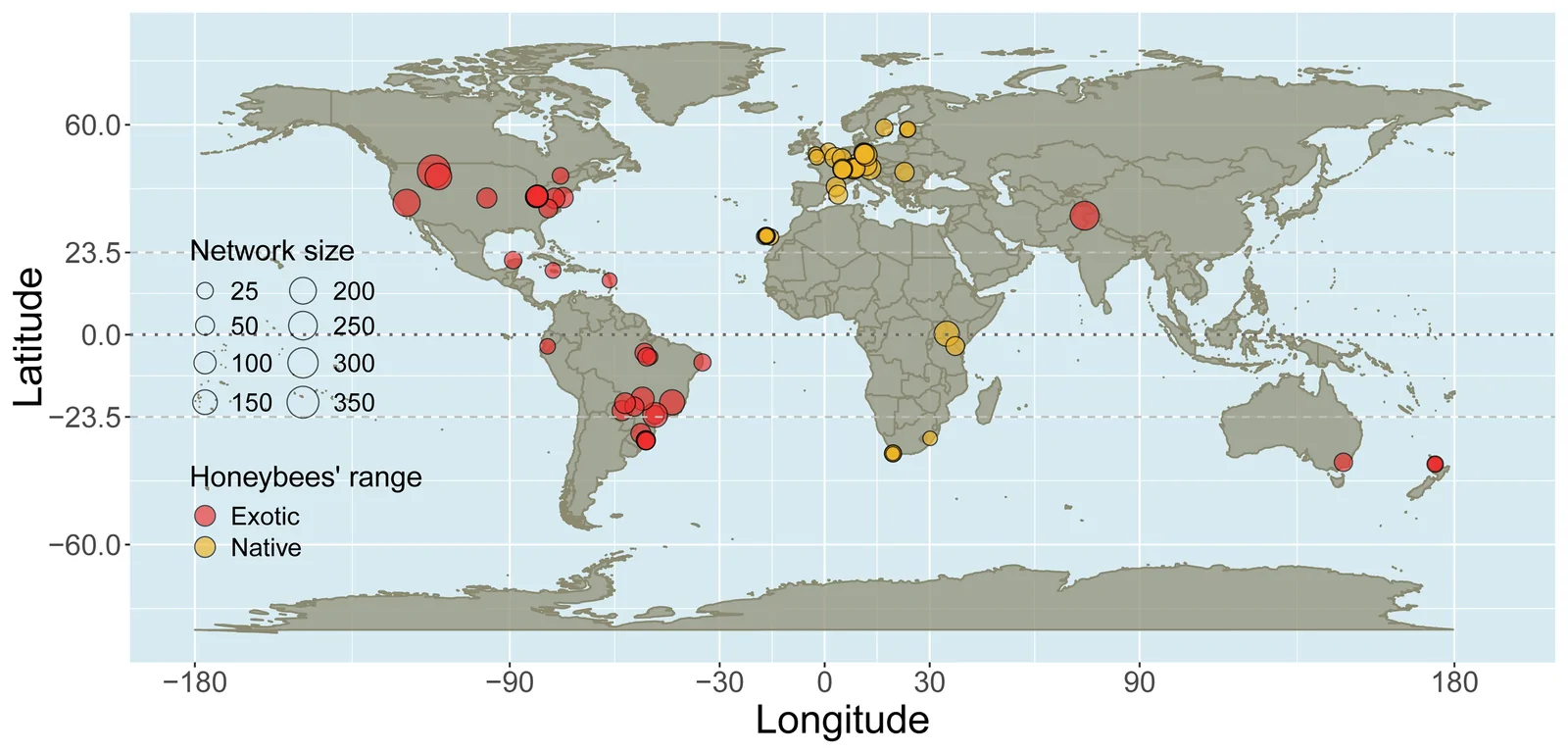
Geographic distribution of the 80 plant–bee interaction networks included in this study across the native (yellow, *n* = 39) and non-native (red, *n* = 41) ranges of *A. mellifera*. Circle size is proportional to network size (number of recorded interactions). The dataset spans a broad geographic extent across all continents except Antarctica, covering a wide range of biogeographic and environmental contexts.

## 2. Methods

### (a) Plant–bee interaction network dataset

Following the Preferred Reporting Items for Systematic Reviews and Meta-Analyses (PRISMA) protocol [27], we compiled a comprehensive dataset of plant–bee interaction networks including *A. mellifera*. We searched the Web of Science database without temporal restrictions (through 2024). The earliest study meeting our inclusion criteria was published in 1976. We supplemented this search with records from Google Scholar, Dryad, Web of Life and studies cited in previous systematic reviews. To ensure a broad and inclusive search, we accounted for both American and British spelling variations by using logical operators (AND/OR). The search string ‘(networ* AND pollina*) OR floral-visit* OR plant–bee*’ captured all relevant variations of the keywords ‘network’, ‘pollinator’, ‘floral-visitor’ and ‘plant–bee’. Our search predominantly included peer-reviewed English-language literature. We relied on internationally indexed databases to ensure a transparent, standardized and reproducible search strategy across regions. Although this approach enhances reproducibility and accessibility, it may underrepresent studies published exclusively in local or regional journals. The initial search produced 1513 records, from which duplicates were removed in accordance with the PRISMA 2020 guidelines [27] (supplementary material, figure S1). We then applied a series of inclusion criteria to select suitable networks for analysis. Eligible networks had to (i) include *A. mellifera* among pollinators, (ii) represent only interactions between plants and bees, (iii) be quantitative (weighted), (iv) be sampled using the phytocentric approach, i.e. recording legitimate pollinator visits on flowers, (v) represent entire communities rather than experimental or focal-species datasets, and (vi) encompass at least one complete flowering season. When the same community was sampled across multiple flowering seasons or years, interaction records were pooled across sampling periods to construct a single quantitative network. Networks collected less than 2 km apart were considered part of the same community and were excluded to avoid spatial pseudoreplication [28,29]. This threshold is consistent with typical foraging distances of many bees, including *A. mellifera* (1–2 km under natural conditions [28,29]). Although dispersal distances vary among bee taxa, this threshold was used as a conservative criterion to reduce spatial pseudoreplication rather than to represent species-specific movement limits. We also removed all pairwise interactions involving animal taxa outside the bee group (Anthophila). To evaluate potential sampling biases, we estimated sampling completeness as the proportion of observed to estimated interaction richness using the Chao1 estimator [30] and tested whether it varied with honeybees' distribution range and network size (i.e. number of plant and bee species). The interaction between range and network size was not significant ($\chi^{2}$ = 0.79, *p* = 0.37), and sample completeness did not differ between native and non-native ranges ($\chi^{2}$ = 0.42, *p* = 0.52; supplementary material, figures S2 and S3).

### (b) Network properties across organizational scales

To assess whether range-dependent patterns emerge at different scales of network organization, we calculated metrics across three organizational levels (figure 2a). At the macro-scale, we compared overall network architecture between ranges by calculating several metrics related to structure, specialization and robustness. We estimated connectance (*C*), the proportion of realized versus potential interactions; bee niche overlap (*R₀*), representing the degree of overlap in plant use among bee species [31,32]; and network-wide specialization (*H_2_′*), quantifying interaction selectivity [33]. Weighted nestedness (*wNODF*) was calculated to describe asymmetrical dependency between specialists and generalists [34,35], while weighted modularity (*Q′*) was used to measure the compartmentalization of networks into internally cohesive modules [36]. Both nestedness and modularity were ∆-transformed to represent deviations from null expectations [37,38], and null distributions were generated using 10 000 randomizations of the *vaznull* algorithm, which preserves network size, species richness and connectance [39]. Finally, we calculated network robustness (*Rd*) as the proportion of bee species persisting after sequential removal of plants, simulating cascading extinctions from least to most connected species [40]. At the macro-scale, these metrics assess whether *A. mellifera* is associated with reorganization of bee–plant networks across ranges. *C* reflects interaction density and redundancy, whereas bee niche overlap captures resource sharing and potential competition among bees. *H_2_′* measures network-wide specialization, with lower values indicating greater generalization. *wNODF* deviations indicate whether *A. mellifera* reinforces hierarchical organization by contributing to interaction patterns in which specialist species interact with a core of highly connected generalists. *Q′* indicates whether *A. mellifera* connects or maintains distinct groups of interacting partners. *Rd* measures whether bee communities become more or less resilient to plant loss following honeybee incorporation.

**Figure 2.**
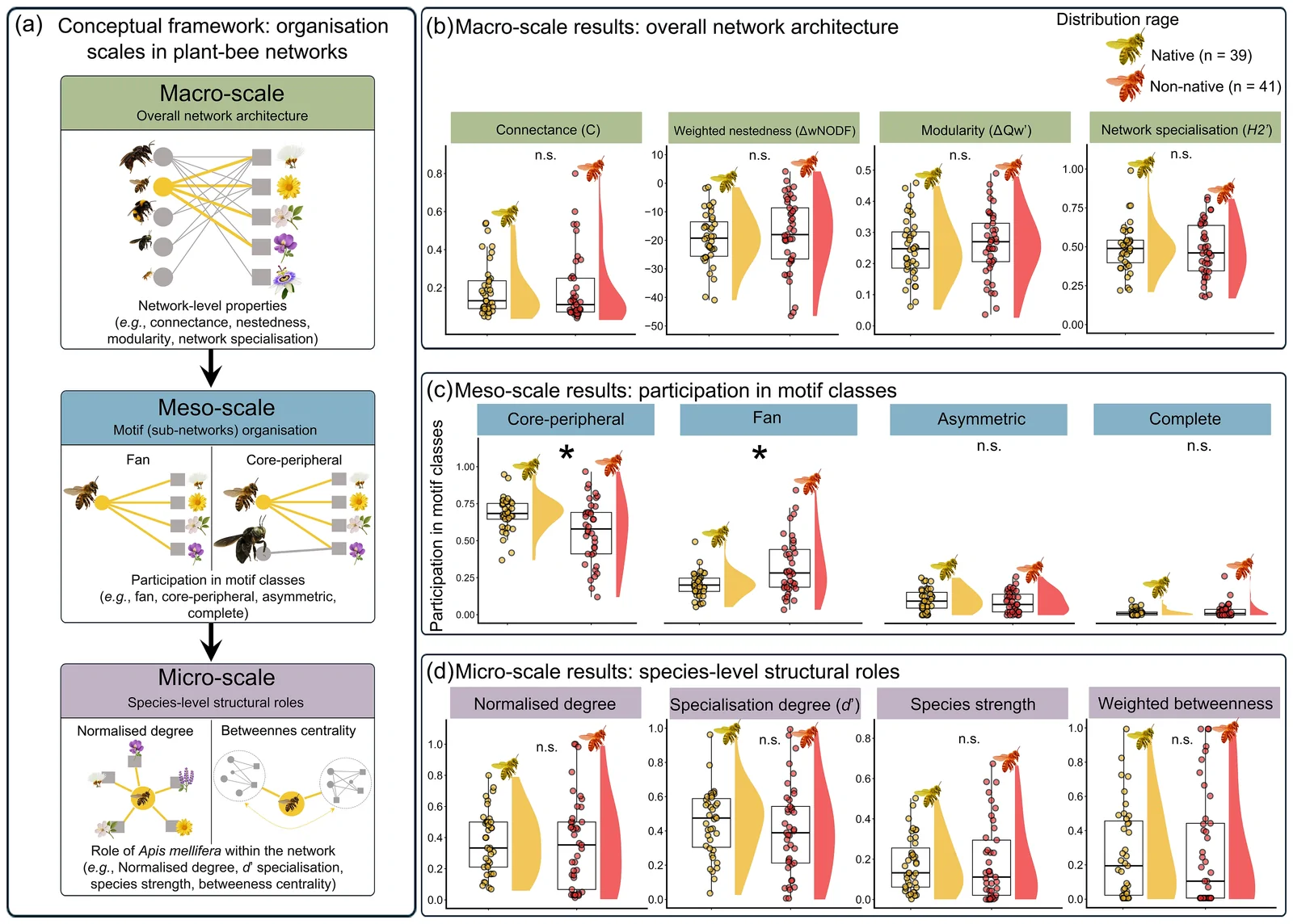
Cross-scale comparison of plant–bee network structure across the native and non-native ranges of *A. mellifera*. Conceptual representation of the three organizational scales analysed in this study (a) and comparison of network properties between the honeybee's native (yellow, *n* = 39) and non-native (red, *n* = 41) ranges. Macro-scale analyses (b) show whole-network properties, including connectance (*C*), weighted nestedness (ΔwNODF), weighted modularity (ΔQw′) and network specialization (*H2*’). Meso-scale analyses (c) show the proportion of motifs involving *A. mellifera* in each motif class, including core–peripheral, fan, asymmetric and complete motifs. Micro-scale analyses (d) show species-level structural roles of *A. mellifera*, including normalized degree, specialization degree (*d′*), species strength and weighted betweenness centrality. Boxplots and points represent observed values across networks, whereas violin plots show density distributions. Asterisks indicate significant differences between ranges (*p* < 0.01), and ‘n.s.’ indicates non-significant differences.

At the meso-scale, we examined the composition of interaction motifs, i.e. small, recurring subnetworks that represent distinct topological arrangements of species interactions [41,42]. Following [43], we classified motifs into four categories according to their connectivity patterns and path lengths: (i) core–peripheral motifs, composed of interconnected generalists linked to one or more specialists; (ii) complete motifs, where all species interact with each other; (iii) asymmetric complete motifs, similar to complete ones but with an attached specialist; (iv) fan motifs, characterized by multiple specialists sharing a single generalist or a single pollinator visiting multiple plants. We quantified motif frequencies using the R package *bmotif* [44] and measured the proportion of each motif type in which *A. mellifera* participated across networks in its native and non-native ranges. For this analysis, we considered three- to six-species motifs and excluded two-species single-pair configurations. This approach allowed us to assess potential shifts in *A. mellifera*'s structural role at the meso-scale structural level. Motif composition captures how *A. mellifera* is integrated into the small, recurring interaction patterns that form the building blocks of ecological networks [45]. Increased participation in fan motifs would indicate that interactions are becoming more centralized around a dominant generalist species, whereas involvement in core–peripheral motifs reflects integration into the most highly connected part of the network [45]. Variation in complete and asymmetric motifs signals changes in how tightly species are linked (i.e. interaction compartmentalization) and how exclusive their interaction partners are (i.e. partner exclusivity [45]). At the micro-scale, we quantified *A. mellifera* roles using complementary metrics that capture interaction breadth, selectivity and functional importance. Normalized degree measures the proportion of available partners used by *A. mellifera*, reflecting its interaction breadth. *d′* quantifies how selectively a species interacts with available partners, with higher values indicating greater interaction selectivity [33,46]. Proportional species strength represents the relative dependence of plant partners on *A. mellifera* compared with other bees in the network. We used the proportional form because species strength is a cumulative metric, and standardization prevents inflation of values with increasing network size [47]. Finally, weighted betweenness centrality reflects the extent to which *A. mellifera* connects otherwise weakly connected parts of the interaction network, indicating its potential role in linking species and facilitating resource flow [48]. Detailed definitions of all metrics are provided in the supplementary material.

### (c) Statistical analysis

To test whether network properties differed between *A. mellifera*'s native and non-native ranges, we fitted generalized linear mixed models using *glmmTMB* in R, with residual diagnostics performed in DHARMa (supplementary material, figures S3, S9–S19; [49]). Network metrics at each organizational scale (macro-, meso- and micro-) were modelled as response variables, with range (native versus non-native) as a fixed effect. Because network size can influence metrics across scales, it was included as a covariate together with its interaction with range. For meso-scale analyses, we additionally included the interaction between range and motif class in the model to assess whether *A. mellifera*'s participation across motif types differed between its native and exotic ranges. Biome was included as a random intercept to account for shared environmental and biogeographic context among networks and to capture non-independence arising from climate, regional species pools and evolutionary history. Error distributions were specified according to the properties of each response variable, and full model specifications are provided in table S1 in the supplementary material. Spatial autocorrelation was assessed using Moran's I tests.

## 3. Results

Our search identified 41 studies that met the inclusion criteria, resulting in a dataset of 80 quantitative plant–bee interaction networks encompassing all continents except Antarctica (figure 1). Among these, 51.25% (*n* = 41) were from the *A. mellifera* non-native range and 48.75% (*n* = 39) from its native range. Across all networks, we documented 1181 plant species and 1047 bee species in the non-native range, compared with 572 plant species and 496 bee species in the native range. On average, *A. mellifera* accounted for a similar proportion of floral visits in its native (26.7 ± 24.1%, mean ± s.d.) and non-native ranges (26.5 ± 28.8%).

Across scales, network structure was largely consistent between the native and non-native ranges of *A. mellifera* (full statistics in tables S2–S5 in the supplementary material). None of the macro-scale network metrics differed between ranges (figure 2b; supplementary material, table S2). Overall, network architecture appears conserved across the honeybees' global distribution (figure 2b; supplementary material, table S2).

At the meso-scale, when focusing on motifs involving *A. mellifera*, clear structural shifts emerged: the species participated less frequently in core–peripheral motifs and more frequently in fan motifs in non-native networks (figure 2c; supplementary material, table S3). This pattern suggests a greater tendency towards resource monopolization within interaction subnetworks outside *A. mellifera*'s native range, despite the absence of broad structural changes at the whole-network level. Further examination of honeybee positions within these motifs showed that fan configurations in which *A. mellifera* occupied the generalist position represented a greater mean proportion of its total motif participation in the non-native than in the native range (21.4 ± 3.9% versus 11.6 ± 1.2%, mean ± SE). Conversely, core–peripheral configurations in which *A. mellifera* occupied a generalist position were more represented in the native than in the non-native range (44.7 ± 3.9% versus 33.2 ± 3.8%; supplementary material, figure S4).

At the micro-scale, honeybees' roles were broadly similar between ranges (figure 2d). No differences were detected in specialization, species strength, betweenness or normalized degree (figure 2d; supplementary material, table S4), indicating that the functional roles of *A. mellifera* within networks remain comparable across regions.

Although some macro-scale metrics, namely, connectance (*C*), bee niche overlap (*R₀*), network-wide specialization (*H_2_′*) and robustness to bee loss (*Rd*), varied with network size, the absence of range × network size interactions indicates that these patterns were not driven by systematic differences in network size between regions (supplementary material, table S2). Similarly, no interactions between range and network size were detected for any meso- and micro-scale metric and no effect of network size alone (supplementary material, tables S3 and S4), reinforcing that observed patterns are not affected by network size (supplementary material, figures S5–S7 and tables S3 and S4). Spatial autocorrelation was detected for some response variables, although effect sizes were generally low to moderate and declined across neighbourhood distances (supplementary material, table S5). Importantly, these patterns did not alter the direction, magnitude or statistical significance of the main effects, indicating that our conclusions were robust to spatial clustering among studies. Hence, these findings together indicate that while the overall architecture and species roles in plant–bee networks remain stable across the global distribution of *A. mellifera*, subtle shifts in motif participation reveal context-dependent changes in how the species integrates into local interaction substructures. Residual diagnosis for each model can be found in figures S8–S18 in the supplementary material.

## 4. Discussion

By examining networks spanning a broad distribution of *A. mellifera* across the globe, we found that plant–bee network structures were broadly convergent across honeybee native and non-native ranges when assessed at the macro- and micro-scales. However, motif composition differed consistently between ranges, with fan motifs being more frequent and core–peripheral motifs less frequent in non-native regions. This pattern suggests that, outside its native range, honeybees are more often embedded in asymmetric interaction structures in which a single pollinator interacts with multiple plant species, potentially reflecting a more dominant pattern of resource use and a stronger influence on local interaction organization. Additionally, fan motifs were more frequent in non-native regions, where *A. mellifera* also occupied the generalist position more often, whereas the opposite pattern occurred in core–peripheral motifs. These position-specific differences reinforce the interpretation that shifts in motif composition reflect greater resource monopolization by honeybees outside their native range. Thus, structural convergence at macro- and micro-scales may mask subtle but consistent reorganization at the motif level, i.e. the meso-scale [24]. These findings suggest that ecological changes associated with honeybee introductions may occur through the interaction reorganization within the building blocks of the networks (i.e. motifs), without cascading major changes in overall network structure or species roles.

These findings emphasize that cross-scale analyses are necessary for detecting the subtle, context-specific effects of invasive species that may remain hidden when attention is restricted to the most commonly used descriptors of network structure. To reduce functional differences among pollinator groups and maximize ecological comparability, we focused on bees, which are phylogenetically closer to honeybees and share more similar functional roles within pollination networks. This focus enabled a more direct assessment of mechanisms associated with honeybee introduction. Nevertheless, other pollinator groups may also experience competitive or facilitative effects that deserve further investigation.

Moreover, because *A. mellifera* is managed within both its native and non-native ranges, management may partly blur ecological differences between them. Our dataset is also geographically uneven, with most networks originating from Europe and the Americas. Although this reflects data availability rather than selective inclusion [50], caution is warranted when extrapolating our findings to underrepresented regions such as Africa, Western Asia and Oceania, which differ in ecological context, climate and land use history. In Africa, for example, this interpretation may be further complicated by the coexistence of *A. mellifera* with other large-bodied bee species, whose contribution to floral resource use and pollination network structure remains poorly understood.

The apparent global consistency in *A. mellifera*'s structural role may reflect long-term ecological integration into non-native communities, although alternative explanations such as convergence in network metrics cannot be excluded. Over time, pollination networks may reorganize around a dominant invader, producing structures that converge towards those found in its native range [51,52]. Such apparent equilibria could obscure the transient effects of competition or displacement that likely occurred during early invasion stages but are rarely captured without pre-invasion baselines [53]. Our findings demonstrate that exploring less commonly studied scales, such as interaction motifs, can reveal patterns that remain undetected by macro- and micro-scale metrics. Rather than summarizing network properties into individual metrics, motif analysis preserves distinct interaction configurations among small groups of species, providing complementary information on resource use and potential competition among species [24,54].

The increased frequency of fan motifs in non-native regions, where multiple species interact indirectly through a single generalist, suggests changes in resource-use patterns and dominance dynamics. Core–peripheral motifs, more common in native ranges, represent dense clusters of interactions among generalists and other species, whereas fan motifs capture systems in which a dominant species monopolizes interactions with many partners [32,43]. Hence, the observed shift towards fan motifs may reflect a transition towards resource monopolization by honeybees, or a compensatory rearrangement of native pollinator behaviour to maintain network connectivity. Although our dataset does not include temporal data to directly assess competitive outcomes, these differences align with previous evidence that *A. mellifera* introductions can reshape the topology of pollination networks [4,11,18,55,56].

Despite these local changes, macro-scale properties such as connectance, nestedness, modularity and robustness remained remarkably stable across the honeybee native and introduced ranges. Such convergence may partly reflect the information lost when complex network structure is summarized by whole-network metrics. Similar metric values can conceal shifts in the distribution and configuration of interactions among species, with potential consequences for network robustness and responses to further perturbations [57]. Importantly, these forms of convergence are not necessarily indicative of system stability. Even when network-level metrics remain unchanged, shifts in how interactions are distributed among species can have profound implications for the long-term resilience and redundancy of pollination systems.

Whether the convergence observed here represents a stable configuration or a transient phase of adjustment remains uncertain. If stable, it may indicate that *A. mellifera* has become functionally integrated into many ecosystems, generating a new equilibrium where its dominance defines network architecture. In this scenario, pollination networks may exhibit a form of ecological homogenization, where structural similarity arises across biogeographic boundaries through the spread of a single generalist species. Conversely, if transient, these networks could still be undergoing reorganization, and their apparent resilience may mask delayed disruptions that manifest only after further losses of native species or resources [18]. The resilience of pollination networks under ongoing global change will therefore depend on whether native species can adapt to, coexist with, or functionally replace *A. mellifera* within these restructured systems. Integrating long-term datasets and historical baselines will be essential to distinguish between stable equilibria and transitional phases and to forecast the persistence of pollination services in an increasingly homogenized world [55,58,59]. Time-series data and reconstructions from museum collections or historical records can reveal how interaction networks respond to land-use intensification, climate change and biological invasions by capturing temporal changes in species composition and interaction turnover [60]. Future research should test whether variation in honeybee interaction dominance, rather than geographic range alone, better predicts patterns of network reorganization across communities over time.

## Supplementary Material



## Data Availability

All data and R code supporting the findings of this study are publicly available in the Figshare repository [61]. Supplementary material is available online [62].
