# Supplementary material for: Meso-scale reorganization emerges despite broad convergence in plant–bee interaction networks across the honeybee's native and non-native ranges
Source: Biol Lett. 2026 Nov 11;22(11):20260436. doi: 10.1098/rsbl.2026.0436 (PMC13752744; doi:10.1098/rsbl.2026.0436)
Supplement: Supplementary file 1 [file rsbl.2026.0436.zip › rsbl.2026.0436/rsbl20260436_si_001.docx]

**Meso-scale reorganisation emerges despite broad convergence in plant-bee interaction networks across the honeybee’s native and non-native ranges**

Leandro Hachuy-Filho^1,2^, Caio S. Ballarin^1,2*^, João Vitor de Alcantara Viana^3^, Pedro A. Lacerda-Barbosa^1,4^, Felipe W. Amorim^1,2*^

^1^ Laboratório de Ecologia da Polinização e Interações – LEPI, Departamento de Biodiversidade e Bioestatística, Instituto de Biociências, Universidade Estadual Paulista “Júlio de Mesquita Filho” (UNESP), Rua Prof. Dr. Antonio Celso Wagner Zanin, CEP 18618-689, Botucatu, São Paulo State, Brazil

^2^ Programa de Pós-graduação em Zoologia, Instituto de Biociências, Universidade Estadual Paulista “Júlio de Mesquita Filho”.

^3^University of Campinas (UNICAMP), Department of Animal Biology, Campinas, São Paulo 13083-862, Brazil

^4^ Programa de Pós-graduação em Biologia Vegetal, Instituto de Biociências, Universidade Estadual Paulista “Júlio de Mesquita Filho”.

***Correspondence:**	Felipe W. Amorim

E-mail address: [felipe.amorim@unesp.br](mailto:felipe.amorim@unesp.br)

Caio S. Ballarin

E-mail address: [csballarin@gmail.com](mailto:csballarin@gmail.com)

**ORCID**

Leandro Hachuy-Filho: 0000-0001-7348-9757

Caio S. Ballarin: 0000-0001-8299-3189

João Vitor de Alcantara Viana: 0000-0002-5249-5362

Pedro A. Lacerda-Barbosa: 0000-0002-8445-8726

Felipe W. Amorim: 0000-0002-6026-0395

**Content**

**Figure S1.** PRISMA 2020 flow diagram for study selection.

**Figure S2.** Comparison of sampling completeness between plant–bee networks from *Apis mellifera*’s native and non-native ranges.

**Figure S3.** Macro-scale network-level metrics for interaction networks including *Apis mellifera* across its native and non-native ranges.

**Figure S4.**

**Figure S5.** Micro-scale species-level network metris depicting *Apis mellifera*’s contribution to network nestedness across honeybees’ native and non-native ranges.

**Figure S6.** Macro-scale network structural properties along the gradient of network size (z-score) for native and exotic honeybee ranges

**Figure S7.** Motif-class frequency along the gradient of network size (z-score) in native and exotic honeybee ranges.

**Figure S8.** Micro-scale network properties along the gradient of network size (z-score) in native and exotic honeybee ranges.

**Figure S9.** DHARMa residual diagnostics for the model of network-level connectance (C; macro-scale metric).

**Figure S10.** DHARMa residual diagnostics for the model of network-level bee niche overlap (R0; macro-scale metric).

**Figure S11.** DHARMa residual diagnostics for the model of network-level specialization degree (H2’; macro-scale metric).

**Figure S12.** DHARMa residual diagnostics for the model of network-level weighted nestedness (wNODF; macro-scale metric).

**Figure S13.** DHARMa residual diagnostics for the model of network-level weighted modularity (Q’; macro-scale metric).

**Figure S14.** DHARMa residual diagnostics for the model of network-level robustness to species loss(Rd; macro-scale metric).

**Figure S15.** DHARMa residual diagnostics for the model of network-level motif class structure (meso-scale).

**Figure S16.** DHARMa residual diagnostics for the model of species-normalised degree (micro-scale).

**Figure S17.** DHARMa residual diagnostics for the model of species-level specialization degree (micro-scale).

**Figure S18.** DHARMa residual diagnostics for the model of proportional species strength (micro-scale).

**Figure S19.** DHARMa residual diagnostics for the model of species-level weighted betweenness (micro-scale).

**Table S1.** Network metrics analysed across spatial scales.

**Table S2.** Effects of honeybee range and network size on the macro-scale structure of plant–bee networks.

**Table S3.** Effects of honeybee range and network size on meso-scale network structure.

**Table S4.** Effects of honeybee range and network size on the macro-scale structure of plant–bee networks.

**Table S5.** Spatial autocorrelation of model residuals across network metrics. Moran’s I.

**Supplementary methods.** Detailed description of network metrics across spatial scales.

**Figure S1. PRISMA 2020 flow diagram for study selection.** Flow diagram showing the process of identifying and selecting studies that reported interaction networks including *Apis mellifera* worldwide. The screening process involved removing duplicates and excluding studies that did not report interaction networks, used binary networks, or did not include *A. mellifera*. A total of 41 studies were included in the review, encompassing 80 pollination networks containing *A. mellifera* within both its native and non-native ranges.

**Figure S2. Sampling completeness of plant–bee interaction networks across *Apis mellifera*’s native and non-native ranges.** (a) Model-predicted relationship between sampling completeness and network size (z-score) for native (yellow) and non-native (red) ranges, with 95% confidence intervals. The interaction between range and network size was not significant (χ² = 0.79, p = 0.37). (b) Comparison of mean sampling completeness between ranges estimated from the model (native: 0.54, 95% CI 0.47–0.62; non-native: 0.57, 95% CI 0.51–0.64). Sampling completeness did not differ between ranges (χ² = 0.42, p = 0.52; “n.s.”, not significant) and was not influenced by network size (χ² = 2.26, p = 0.13). Variation explained by biome (the random effect) was negligible (<0.01; ~0% ICC).

**Figure S3. DHARMa residual diagnostics for the model of network-level sampling completeness (macros-scale metric).** The upper-left panel shows a quantile–quantile (QQ) plot comparing observed and expected residual distributions, indicating agreement with model assumptions. The upper-right panel displays residuals against fitted values, with quantile regression lines used to detect systematic deviations from uniformity. The lower-left panel presents the dispersion test, comparing observed residual variance with simulations from the fitted model. The lower-right panel shows the distribution of residuals and the outlier test.

**Figure S4. Position-specific participation of *Apis mellifera* in fan and core-peripheral motifs across its native and non-native ranges.** Bars show the mean proportion of total *A. mellifera* participation across the meso-scale motif classes corresponding to configurations in which *A. mellifera* occupied the generalist position within fan or core-peripheral motifs. Error bars represent ± SE across networks (native: n = 39; non-native: n = 40).

**Figure S5. Macro-scale network-level metrics for interaction networks including *Apis mellifera* across its native and non-native ranges.** (a) Comparison of mean connectance (*C*) between ranges estimated from the model (native: 0.12, 95% CI 0.08–0.18; non-native: 0.18, 95% CI 0.14–0.22). Connectance did not differ between ranges (χ² = 3.80, p = 0.051; “n.s.”). Variation explained by biome (the random effect) was minimal (0.02; ~1.5% ICC). (b) Comparison of mean robustness between ranges estimated from the model (native: 0.80, 95% CI 0.75–0.84; non-native: 0.78, 95% CI 0.74–0.82). Robustness did not differ between ranges (χ² = 0.40, p = 0.53; “n.s.”). Variation explained by biome was negligible (0.01; ~0.7% ICC).

**Figure S6. Macro-scale network structural properties along the gradient of network size (z-score) for native and exotic honeybee ranges.** Variation in network-level metrics as a function of standardized network size (z-score) for networks within the native (yellow) and exotic (red) ranges of honeybees. Shaded areas represent confidence intervals around model predictions. Across all metrics, there was no significant interaction between network size and honeybee range, indicating that changes in network structure along the network size gradient followed similar trajectories in both ranges (a; Connectance – *C* – χ² = 1.53, p = 0.22; b; bee niche overlap – *R_0_* – χ² = 0.62, p = 0.43; c; network-level specialisation – *H_2_'* – χ² = 1.78, p = 0.18; d; Weighted nestedness – *ΔwNODF* – χ² = 0.89, p = 0.34; e; weighted modularity – *ΔQw* – χ² = 0.15, p = 0.70; f; robustness to species loss – *R* – χ² = 3.34, p = 0.07). Overall, the absence of significant interaction terms indicates that the effect of network size on network structural properties is consistent across native and exotic honeybee ranges.

**Figure S7. Motif-class frequency along the gradient of network size (z-score) in native and exotic honeybee ranges.** Variation in the relative frequency of four motif classes (asymmetric, complete, core–peripheral, and fan) as a function of standardized network size (z-score) for networks in the native and exotic honeybee ranges. Shaded areas represent confidence intervals around model predictions. Motif class did not interact with network size (χ² = 1.95, p = 0.583), and there was no significant interaction between range and network size (χ² = 1.61, p = 0.205). The three-way interaction among motif class, range, and network size was also not significant (χ² = 1.77, df = 3, p = 0.621). Overall, although motif composition differs between native and exotic ranges, the way motif frequencies vary along the network size gradient is statistically similar between ranges, as indicated by the absence of significant interactions involving network size.

**Figure S8. Micro-scale network properties along the gradient of network size (z-score) in native and exotic honeybee ranges.** Variation in species-level (micro-scale) network metrics as a function of standardized network size (z-score) for networks in the native (yellow) and exotic (red) honeybee ranges. Shaded areas represent confidence intervals around model predictions. For all metrics, the interaction between network size and range was not statistically significant, indicating that species-level responses to increasing network size follow similar trajectories in both ranges (a; Normalised degree – χ² = 0.90, p = 0.34; b; species specialization – d′ – χ² < 0.01, p = 0.98; c) proportional species strength – χ² = 3.15, p = 0.08; d; weighted betweenness centrality – χ² = 3.53, p = 0.06). Overall, the absence of significant interaction terms indicates that the effect of network size on species-level structural properties is consistent across native and exotic honeybee ranges, despite baseline differences in some metrics.

**Figure S9. DHARMa residual diagnostics for the model of network-level connectance (*C*; macro-scale metric).** The upper-left panel shows a quantile–quantile (QQ) plot comparing observed and expected residual distributions, indicating agreement with model assumptions. The upper-right panel displays residuals against fitted values, with quantile regression lines used to detect systematic deviations from uniformity. The lower-left panel presents the dispersion test, comparing observed residual variance with simulations from the fitted model. The lower-right panel shows the distribution of residuals and the outlier test.

**Figure S10. DHARMa residual diagnostics for the model of network-level bee niche overlap (*R_0_*; macro-scale metric).** The upper-left panel shows a quantile–quantile (QQ) plot comparing observed and expected residual distributions, indicating agreement with model assumptions. The upper-right panel displays residuals against fitted values, with quantile regression lines used to detect systematic deviations from uniformity. The lower-left panel presents the dispersion test, comparing observed residual variance with simulations from the fitted model. The lower-right panel shows the distribution of residuals and the outlier test.

**Figure S11. DHARMa residual diagnostics for the model of network-level specialization degree (*H_2_’*; macro-scale metric).** The upper-left panel shows a quantile–quantile (QQ) plot comparing observed and expected residual distributions, indicating agreement with model assumptions. The upper-right panel displays residuals against fitted values, with quantile regression lines used to detect systematic deviations from uniformity. The lower-left panel presents the dispersion test, comparing observed residual variance with simulations from the fitted model. The lower-right panel shows the distribution of residuals and the outlier test.

**Figure S12. DHARMa residual diagnostics for the model of network-level weighted nestedness (*wNODF*; macro-scale metric).** The upper-left panel shows a quantile–quantile (QQ) plot comparing observed and expected residual distributions, indicating agreement with model assumptions. The upper-right panel displays residuals against fitted values, with quantile regression lines used to detect systematic deviations from uniformity. The lower-left panel presents the dispersion test, comparing observed residual variance with simulations from the fitted model. The lower-right panel shows the distribution of residuals and the outlier test.

**Figure S13. DHARMa residual diagnostics for the model of network-level weighted modularity (*Q’*; macro-scale metric).** The upper-left panel shows a quantile–quantile (QQ) plot comparing observed and expected residual distributions, indicating agreement with model assumptions. The upper-right panel displays residuals against fitted values, with quantile regression lines used to detect systematic deviations from uniformity. The lower-left panel presents the dispersion test, comparing observed residual variance with simulations from the fitted model. The lower-right panel shows the distribution of residuals and the outlier test.

**Figure S14. DHARMa residual diagnostics for the model of network-level robustness to species loss(*R_d_*; macro-scale metric).** The upper-left panel shows a quantile–quantile (QQ) plot comparing observed and expected residual distributions, indicating agreement with model assumptions. The upper-right panel displays residuals against fitted values, with quantile regression lines used to detect systematic deviations from uniformity. The lower-left panel presents the dispersion test, comparing observed residual variance with simulations from the fitted model. The lower-right panel shows the distribution of residuals and the outlier test.

**Figure S15. DHARMa residual diagnostics for the model of network-level motif class structure (meso-scale).** The upper-left panel shows the quantile–quantile (QQ) plot comparing observed and expected residual distributions. The upper-right panel displays residuals against fitted values with quantile regression lines to detect systematic deviations. The lower-left panel presents the dispersion test comparing observed and simulated residual variance, and the lower-right panel shows the residual distribution and outlier test.

**Figure S16. DHARMa residual diagnostics for the model of species-normalised degree (micro-scale).** The upper-left panel shows the QQ plot comparing observed and expected residual distributions. The upper-right panel displays residuals versus fitted values to assess potential systematic patterns. The lower-left panel presents the dispersion test contrasting observed and simulated variance, and the lower-right panel shows the residual histogram with the outlier test. Overall, diagnostics indicate adequate model fit with no strong violations of assumptions.

**Figure S17. DHARMa residual diagnostics for the model of species-level specialization degree (micro-scale).** The upper-left panel shows the QQ plot comparing observed and expected residual distributions. The upper-right panel displays residuals versus fitted values to assess potential systematic patterns. The lower-left panel presents the dispersion test contrasting observed and simulated variance, and the lower-right panel shows the residual histogram with the outlier test. Overall, diagnostics indicate adequate model fit with no strong violations of assumptions.

**Figure S18. DHARMa residual diagnostics for the model of proportional species strength (micro-scale).** The upper-left panel shows the QQ plot comparing observed and expected residual distributions. The upper-right panel displays residuals versus fitted values to assess potential systematic patterns. The lower-left panel presents the dispersion test contrasting observed and simulated variance, and the lower-right panel shows the residual histogram with the outlier test. Overall, diagnostics indicate adequate model fit with no strong violations of assumptions.

**Figure S19. DHARMa residual diagnostics for the model of species-level weighted betweenness (micro-scale).** The upper-left panel shows the QQ plot comparing observed and expected residual distributions. The upper-right panel displays residuals versus fitted values to assess potential systematic patterns. The lower-left panel presents the dispersion test contrasting observed and simulated variance, and the lower-right panel shows the residual histogram with the outlier test. Overall, diagnostics indicate adequate model fit with no strong violations of assumptions.

| **Metric** | **Ecological level** | **Model structure** | **Error distribution** |
| --- | --- | --- | --- |
| **Macro-scale** |  |  |  |
| Sampling completeness | Network | response ~ range * network size _(z-score)_ | (1\|biome) |
| Connectance | Network | response ~ range * network size _(z-score)_ | (1\|biome) |
| Bee niche overlap | Guild | response ~ range * network size _(z-score)_ | (1\|biome) |
| H2′ specialization | Network | response ~ range * network size _(z-score)_ | (1\|biome) |
| Δ weighted nestedness | Network | response ~ range * network size _(z-score)_ | (1\|biome) |
| Δ weighted modularity | Network | response ~ range * network size _(z-score)_ | (1\|biome) |
| Robustness | Network | response ~ range * network size _(z-score)_ | (1\|biome) |
| **Meso-scale** |  |  |  |
| Motif class proportion | Subnetwork pattern | proportion ~ class * range * network size _(z-score)_ | (1\|biome) |
| **Micro-scale** |  |  |  |
| d′ specialisation | Species | response ~ range * network size _(z-score)_ | (1\|biome) |
| Normalized degree | Species | response ~ range * network size _(z-score)_ | (1\|biome) |
| Proportional species strength | Species | response ~ range * network size _(z-score)_ | (1\|biome) |
| Weighted betweenness | Species | response ~ range * network size _(z-score)_ | (1\|biome) |

**Table S1. Network metrics analysed across spatial scales.** Summary of all response variables quantified at macro-, meso-, and micro-scales, indicating the ecological level represented by each metric and the statistical model structure used for hypothesis testing. All models included range (native vs. non-native), standardized network size, and their interaction as fixed effects, with biome included as a random intercept.

**Table S2. Effects of honeybee range and network size on the macro-scale structure of plant–bee networks.** Estimated marginal means (± 95% confidence intervals) for native and exotic honeybee ranges are shown for each network metric. Test statistics correspond to Type III Wald χ² tests from generalized linear mixed models including range, network size, and their interaction as fixed effects, and biome as a random intercept. Var(Biome) and the intraclass correlation coefficient (ICC) represent the variance component associated with differences among biomes. Overall, network size was the most consistent predictor of network structure. Honeybee range showed no statistically significant effects across metrics. Interaction terms were not significant for any response variable, suggesting that the effect of network size on network structure does not differ between native and exotic ranges. Variation attributable to biome was generally small, suggesting that most structural variation occurred within rather than among biomes.

| **Metric** | **Distribution** | **Mean native (95% CI)** | **Mean exotic (95% CI)** | **χ² Range (P)** | **χ² Network size (P)** | **χ² Range : Network size (P)** | **Var (Biome / ICC)** |
| --- | --- | --- | --- | --- | --- | --- | --- |
| **Connectance** | Beta  (cloglog) | 0.12  (0.08–0.18) | 0.18  (0.14–0.22) | 3.80 (0.051) | ***24.17***  ***(<0.01)*** | 1.53  (0.22) | 0.02  (~1.5%) |
|  |  |  |  |  |  |  |  |
| **Bee niche overlap** | Beta  (cloglog) | 0.15  (0.09–0.23) | 0.19  (0.15–0.25) | 1.80  (0.18) | ***4.77***  ***(0.03)*** | 0.62  (0.43) | 0.04  (~2.7%) |
|  |  |  |  |  |  |  |  |
| **H_2_’ specialisation** | Gaussian (identity) | 0.51  (0.42–0.60) | 0.49  (0.41–0.57) | 0.47  (0.49) | ***4.29***  ***(0.04)*** | 1.78  (0.18) | 0.01  (~26.5%) |
|  |  |  |  |  |  |  |  |
| **ΔwNODF** | Gaussian (identity) | -21.3  (-26.3 to -16.2) | -20.5  (-24.9 to -16.0) | 0.10  (0.75) | <0.01  (0.98) | 0.89  (0.34) | 13.06  (~11%) |
|  |  |  |  |  |  |  |  |
| **ΔModularity** | Gaussian (identity) | 0.26  (0.22–0.29) | 0.26  (0.23–0.30) | 0.07  (0.80) | 0.53  (0.47) | 0.15  (0.70) | <0.01  (~1.9%) |
|  |  |  |  |  |  |  |  |
| **Robustness** | Beta  (cloglog) | 0.80  (0.75–0.84) | 0.78  (0.74–0.82) | 0.40  (0.53) | ***16.57***  ***(<0.01)*** | 3.34  (0.07) | 0.01  (~0.7%) |

**Table S3. Effects of honeybee range and network size on meso-scale network structure.** Results from a beta GLMM with logit link testing differences in the relative frequency of motif classes across honeybee ranges and network size. Wald χ² tests evaluate fixed effects, with biome included as a random intercept; the variance component (Var(biome) <0.01) and ICC ~ 0% indicate a negligible biome effect. Estimated marginal means (±95% CI) are shown. Motif composition differed strongly among classes, and the significant class × range interaction indicates that range effects depend on motif type, with core motifs more frequent in native ranges and fan motifs more frequent in exotic ranges, while network size had no significant effect.

| **Effect** | | **χ²** | **df** | **P** |
| --- | --- | --- | --- | --- |
| Motif class | | 409.94 | 3 | ***<0.001*** |
| Range | | 1.10 | 1 | 0.294 |
| Network size | | 2.24 | 1 | 0.134 |
| Motif class × Range | | 17.85 | 3 | ***<0.001*** |
| Motif class × Network size | | 1.95 | 3 | 0.583 |
| Range × Network size | | 1.61 | 1 | 0.205 |
| Motif class × Range × Network size | | 1.77 | 3 | 0.621 |
| Distribution | | Beta (logit) | — | — |
| Var(Biome / ICC) | | <0.01 (~0%) | — | — |
| Marginal R² | | 0.883 | — | — |
| **Motif class × Range comparison** | | | | |
| **Motif class** | **Mean native (95% CI)** | **Mean exotic (95% CI)** | | **P** |
| Asymmetric | 0.104 (0.080–0.135) | 0.086 (0.066–0.112) | | 0.29 |
| Complete | 0.041 (0.029–0.057) | 0.044 (0.032–0.059) | | 0.80 |
| Core-peripheral | ***0.672 (0.620–0.719)*** | ***0.569 (0.520–0.617)*** | | ***<0.01*** |
| Fan | ***0.219 (0.179–0.265)*** | ***0.313 (0.270–0.360)*** | | ***<0.01*** |

**Table S4. Effects of honeybee range and network size on the micro-scale structure of plant–bee networks**. Test statistics correspond to Type III Wald χ² tests from generalized linear mixed models including range, network size, and their interaction as fixed effects, and biome as a random intercept. Var(Biome) and the intraclass correlation coefficient (ICC) represent the variance component associated with differences among biomes. Honeybee range showed no statistically significant effects across species level (*Apis mellifera*) metrics. Network size showed a positive effect on weighted betweenness. Interaction terms were not significant for any response variable, suggesting that the effect of network size on network structure does not differ between native and exotic ranges. Variation attributable to biome was generally small, suggesting that most structural variation occurred within rather than among biomes.

| **Metric** | **Distribution** | **Mean native (95% CI)** | **Mean exotic (95% CI)** | **χ² Range (P)** | **χ² Network size (P)** | **χ² Range : Network size (P)** | **Var (Biome / ICC)** |
| --- | --- | --- | --- | --- | --- | --- | --- |
| Normalised degree | Gaussian (identity) | 0.42  (0.31–0.54) | 0.41  (0.31–0.51) | 0.06 (0.80) | 0.20  (0.65) | 0.90  (0.34) | <0.01  (~9.5%) |
|  |  |  |  |  |  |  |  |
| Specialisation *d*′ | Gaussian (identity) | 0.41  (0.32–0.51) | 0.50  (0.38–0.62) | 1.72 (0.19) | 0.47  (0.49) | <0.01  (0.98) | <0.01  (~12.5%) |
|  |  |  |  |  |  |  |  |
| Proportional species strength | Tweedie  (logit) | 0.27  (0.14–0.46) | 0.20  (0.13–0.30) | 0.98 (0.32) | 1.33  (0.25) | 3.15  (0.08) | 0.22  (~25.3%) |
|  |  |  |  |  |  |  |  |
| Weighted betweenness | Beta  (logit) | 0.40  (0.26–0.56) | 0.32  (0.22–0.45) | 1.29 (0.26) | 4.43  (0.03) | 3.53  (0.06) | 0.10  (~13.2%) |

**Table S5. Spatial autocorrelation of model residuals across network metrics. Moran’s I.** statistics calculated on model residuals using k-nearest neighbour spatial weights (k = 4–14). Values are reported as Moran’s I with associated permutation p-values. Most metrics showed low or non-significant autocorrelation, indicating substantial spatial independence of residuals. Significant but generally moderate positive autocorrelation occurred mainly at small spatial neighbourhoods for some structural metrics (e.g., connectance and niche overlap), and declined with increasing k. The motif analysis for *Apis mellifera* showed no spatial autocorrelation (I ~ 0), supporting that observed range effects are not driven by spatial structure.

| **Metric** | **k=4** | **k=6** | **k=8** | **k=10** | **k=12** | **k=14** |
| --- | --- | --- | --- | --- | --- | --- |
| **Macro-scale** |  |  |  |  |  |  |
| Sampling completeness | 0.07 (0.11) | 0.07 (0.07) | 0.03 (0.21) | <0.01 (0.33) | -0.02 (0.60) | -0.05 (0.84) |
| Connectance | 0.22 (<0.01) | 0.14 (<0.01) | 0.13 (<0.01) | 0.09 (<0.01) | 0.06 (0.03) | 0.05 (0.02) |
| Bee niche overlap | 0.31 (<0.01) | 0.29 (<0.01) | 0.24 (<0.01) | 0.20 (<0.01) | 0.15 (<0.01) | 0.13 (<0.01) |
| Specialisation H_2_’ | 0.28 (<0.01) | 0.15 (<0.01) | 0.11 (0.05) | 0.10 (<0.01) | 0.04 (0.08) | 0.04 (0.06) |
| ΔWNODF | 0.06 (0.15) | 0.07 (0.06) | <0.01 (0.38) | -0.05 (0.80) | -0.05 (0.87) | -0.07 (0.94) |
| ΔModularity | <0.01 (0.42) | 0.02 (0.29) | <0.01 (0.37) | -0.03 (0.64) | -0.03 (0.64) | -0.01 (0.48) |
| Robustness | 0.10 (0.05) | 0.02 (0.29) | 0.02 (0.24) | 0.01 (0.26) | 0.01 (0.25) | -<0.01 (0.36) |
| **Meso-scale** |  |  |  |  |  |  |
| Motifs | -0.24 (0.99) | -0.15 (0.99) | -0.11 (0.99) | -0.08 (0.99) | -0.07 (0.99) | -0.06 (0.99) |
| **Micro-scale** |  |  |  |  |  |  |
| Normalised degree | 0.24 (<0.01) | 0.22 (<0.01) | 0.17 (<0.01) | 0.12 (<0.01) | 0.08 (<0.01) | 0.05 (0.03) |
| Specialisation d′ | -0.01 (0.51) | -0.01 (0.49) | <0.01 (0.37) | 0.02 (0.21) | -0.01 (0.46) | 0.01 (0.24) |
| Proportional species strength | 0.37 (<0.01) | 0.29 (<0.01) | 0.25 (<0.01) | 0.19 (<0.01) | 0.14 (<0.01) | 0.09 (0.01) |
| Weighted betweenness | 0.14 (0.01) | 0.15 (<0.01) | 0.13 (<0.01) | 0.11 (<0.01) | 0.08 (<0.01) | 0.04 (0.06) |

**Supplementary Methods -** Detailed description of network metrics across spatial scales.

Macro-scale metrics (whole-network structure)

Macro-scale metrics describe emergent structural properties of entire plant–pollinator networks and capture how interaction architecture is organized at the community level.

***Sampling completeness***

Sampling completeness was included to ensure that differences in network structure were not artifacts of unequal interaction detection. Network metrics are sensitive to sampling intensity, particularly connectance and specialization (Chacoff et al. 2012). Including this metric ensures comparability across regions.

*Calculation:* We estimated completeness using coverage-based nonparametric estimators derived from frequency data following the framework developed by Chao (1984) and later extended for diversity and interaction data (Chao et al. 2014; Hsieh et al. 2016). These estimators infer asymptotic interaction richness based on the frequency of rare interactions (singletons and doubletons), which contain information about undetected links. For each network, interaction richness was estimated using Chao-type estimators that incorporate the number of singletons (interactions observed once) and doubletons (observed twice). Estimated asymptotic richness was then compared to observed richness to obtain a proportional completeness value. Values approaching 1 indicate near-exhaustive sampling, whereas lower values indicate potential under-detection of rare interactions

***Connectance***

Connectance is defined as the proportion of realized interactions relative to all possible interactions between plants (I) and pollinators (J). It is calculated as the number of observed links divided by I × J (Pascual & Dunne 2006). Connectance is a foundational descriptor of ecological network complexity and has direct implications for stability theory. In mutualistic systems, higher connectance is often associated with increased redundancy and buffering capacity against species loss, although it may also intensify indirect effects.

*Calculation:* Connectance was calculated from the quantitative interaction matrix after converting it to a binary matrix (presence–absence of interactions). The total number of realized links was divided by the total number of possible plant–pollinator combinations within each network.

***Bee niche overlap (Horn index)***

Bee niche overlap was quantified using the Horn index (Horn 1966), a quantitative similarity measure derived from Shannon entropy and appropriate for interaction frequency data. The Horn index measures proportional similarity in resource use between pairs of species while incorporating interaction weights. It is less sensitive to rare interactions than some alternative indices and performs well for abundance-based bipartite data. High average Horn overlap among bee species indicates greater sharing of floral resources, suggesting reduced niche partitioning and potentially stronger exploitative competition. In invasion contexts, increased overlap may reflect functional homogenization driven by a dominant generalist species.

*Calculation:* Pairwise niche overlap between bee species was calculated using the Horn index based on quantitative interaction frequencies. For each network, proportional interaction frequencies were computed for each bee species across plant partners, and pairwise similarity values were obtained. The network-level metric corresponds to the mean pairwise Horn overlap among bee species within each network.

***Network-level specialisation (H_2_′)***

H_2_′ is a quantitative specialisation index derived from Shannon entropy and standardised between theoretical minimum and maximum values (Blüthgen et al. 2006). It measures how selectively species interact relative to partner availability across the entire bipartite network. Because it incorporates interaction frequencies and corrects for marginal totals, H_2_′ distinguishes true interaction specialisation from patterns driven by abundance. Higher H_2_′ indicates structured specialisation; lower values indicate generalised interaction architecture.

*Calculation:* H_2_′ was calculated using the quantitative interaction matrix, incorporating interaction frequencies and correcting for marginal totals. The metric standardizes Shannon entropy of interactions between theoretical minimum and maximum values given the observed row and column totals, allowing comparison across networks with different sizes and interaction totals

***Weighted nestedness (wNODF)***

Nestedness describes hierarchical organization in which specialist species interact with subsets of partners used by generalists. We used the weighted NODF metric (Almeida-Neto et al. 2008), which incorporates interaction frequencies. Nested organization has been proposed as a stabilizing feature of mutualistic networks (Bascompte et al. 2003). To control for size- and density-dependent biases, we analysed deviation from null model expectations (Δ metric), isolating structural organization beyond random assembly processes.

*Calculation:* The wNODF metric is based on two principles: i) Decreasing marginal totals: rows and columns are ordered by decreasing interaction totals. ii) Paired overlap: for each ordered pair of species, the metric quantifies how much the less-connected species’ interaction pattern overlaps with that of the more-connected species. In the weighted version, overlap is computed using quantitative interaction values rather than simple presence–absence. Nestedness is then averaged across all valid row and column pairs to produce a network-level index.

***Weighted modularity (Qw)***

Modularity quantifies the degree to which networks are partitioned into modules (i.e., subsets of species interacting more strongly among themselves than with others). We used weighted modularity for bipartite networks as described by Dormann & Strauss (2014). Modular structure may buffer perturbations by containing disturbances within compartments, but it may also reflect ecological differentiation or coevolutionary structuring. As with nestedness, we analysed deviation from null expectations to avoid confounding effects of network size.

*Calculation:* We calculated weighted modularity using the DIRTLPAwb+ algorithm (Beckett 2016), specifically developed for quantitative bipartite networks. DIRTLPAwb+ (Deterministic Iterative Refinement of the Label Propagation Algorithm for weighted bipartite networks) works as follows: i) Species are initially assigned to modules. ii) Labels (module identities) are iteratively updated to maximize modularity gain. iii) A deterministic refinement step improves partition quality. iv) The “+” extension improves convergence and avoids local optima. This algorithm directly incorporates interaction weights and is computationally efficient for large bipartite matrices.

***Robustness to species loss***

Robustness was estimated as the area under the secondary extinction curve following sequential plant removal (Memmott et al. 2004). This metric quantifies structural tolerance to disturbance and represents expected persistence of pollinators following plant loss. Higher robustness indicates greater redundancy and stability.

*Calculation:* Following the framework proposed by Memmott et al. (2004) and formalized quantitatively by Burgos et al. (2007), plant species were sequentially removed (downward extinction sequence). After each removal step, pollinators lacking interaction partners were considered secondarily extinct. The cumulative proportion of extinct pollinators was plotted against the proportion of removed plants, generating an extinction curve. Robustness was calculated as the area under this curve (AUC), which was standardized to range between 0 and 1 (Burgos et al. 2007). Values close to 1 indicate gradual secondary extinction (high tolerance), whereas values close to 0 indicate rapid collapse. This standardization allows direct comparison across networks differing in size.

Meso-scale metrics (motif structure involving *Apis mellifera*)

Motifs are small recurring interaction configurations that serve as building blocks of ecological networks. We quantified the proportional representation of motif classes following Simmons et al. (2019, 2021).

**Fan motifs** represent centralized interaction patterns in which multiple species share a common partner. **Core–peripheral** motifs reflect asymmetric clustering with highly connected “core” species and peripheral specialists. **Complete** motifs represent fully interconnected subsets of species, indicating dense reciprocal interaction clusters. **Asymmetric complete** motifs reflect imbalanced participation within otherwise cohesive substructures. Motif composition provides intermediate-scale insight between species-level roles and whole-network organization, revealing subtle reorganization patterns that global metrics may not detect.

*Calculation:* Motif frequencies were calculated by enumerating all possible three- and four-species subgraphs in each quantitative bipartite network and classifying them into predefined motif categories. For each network, counts of each motif class were divided by the total number of motifs to obtain proportional representation. These proportions were used as response variables.

Micro-scale metrics (species-level roles of *Apis* *mellifera* in each network)

Micro-scale metrics quantify the structural role of individual species within networks.

***Normalised degree***

Normalised degree represents the proportion of available partners with which a species interacts (Pascual & Dunne 2006). It captures interaction breadth while controlling for network size.

*Calculation:* Normalised degree was calculated as the number of plant partners visited by the focal species divided by the total number of plant species available within the network. This standardization removes direct dependence on network size.

***d′ specialisation***

The species-level specialisation index d′ is derived from the standardised Kullback–Leibler divergence between observed and expected partner use (Blüthgen et al. 2006). It measures how selectively a species interacts relative to partner availability. Values near zero indicate opportunistic generalisation; values near one indicate strong selectivity.

*Calculation:* Species-level d′ was calculated from quantitative interaction matrices by comparing observed interaction frequencies of the focal species to expected frequencies under random partner use proportional to availability. The resulting divergence was standardized to range between 0 and 1.

***Proportional species strength***

Species strength quantifies the cumulative dependence of partner species on a focal species and was formalized for quantitative mutualistic networks by Bascompte et al. (2006). Using proportional species strength controls for variation in network size and total interaction frequency. High values indicate asymmetric dependence and potential ecological dominance.

*Calculation:* Species strength was first calculated as the sum of dependencies of plant species on the focal pollinator, where plant dependence corresponds to the proportion of its total interactions attributable to that pollinator. To account for differences in network size and interaction totals, species strength was then expressed proportionally within each network before statistical analysis.

***Weighted betweenness centrality***

Weighted betweenness centrality measures how frequently a species lies on shortest weighted paths between other species (Newman 2010). Species with high betweenness act as structural connectors linking otherwise weakly connected subgroups and may influence cohesion and disturbance propagation across modules.

*Calculation:* Weighted betweenness was computed using the quantitative interaction matrix by transforming interaction frequencies into edge weights and calculating shortest weighted paths between all pairs of species in the projected network. Betweenness values were standardized within networks prior to modelling to allow comparability across networks of different sizes.

**References**

Almeida‐Neto, M., Guimaraes, P., Guimaraes Jr, P. R., Loyola, R. D., & Ulrich, W. (2008). A consistent metric for nestedness analysis in ecological systems: reconciling concept and measurement. Oikos, 117(8), 1227-1239.

Bascompte, J., Jordano, P., Melián, C. J., & Olesen, J. M. (2003). The nested assembly of plant–animal mutualistic networks. Proceedings of the National Academy of Sciences, 100(16), 9383-9387.

Bascompte, J., Jordano, P., & Olesen, J. M. (2006). Asymmetric coevolutionary networks facilitate biodiversity maintenance. Science, 312(5772), 431-433.

Beckett, S. J. (2016). Improved community detection in weighted bipartite networks. Royal Society open science, 3(1).

Blüthgen, N., Menzel, F., & Blüthgen, N. (2006). Measuring specialization in species interaction networks. BMC ecology, 6(1), 9.

Burgos, E., Ceva, H., Perazzo, R. P., Devoto, M., Medan, D., Zimmermann, M., & Delbue, A. M. (2007). Why nestedness in mutualistic networks?. Journal of theoretical biology, 249(2), 307-313.

Chacoff, N. P., Vázquez, D. P., Lomáscolo, S. B., Stevani, E. L., Dorado, J., & Padrón, B. (2012). Evaluating sampling completeness in a desert plant–pollinator network. Journal of Animal Ecology, 81(1), 190-200.

Chao, A. (1984). Nonparametric estimation of the number of classes in a population. Scandinavian Journal of statistics, 265-270.

Chao, A., Gotelli, N. J., Hsieh, T. C., Sander, E. L., Ma, K. H., Colwell, R. K., & Ellison, A. M. (2014). Rarefaction and extrapolation with Hill numbers: a framework for sampling and estimation in species diversity studies. Ecological monographs, 84(1), 45-67.

Dormann, C. F., & Strauss, R. (2014). A method for detecting modules in quantitative bipartite networks. Methods in Ecology and Evolution, 5(1), 90-98.

Hsieh, T. C., Ma, K., & Chao, A. (2016). iNEXT: an R package for rarefaction and extrapolation of species diversity (H ill numbers). Methods in ecology and evolution, 7(12), 1451-1456.

Horn, H. S. (1966). Measurement of "overlap" in comparative ecological studies. The American Naturalist, 100(914), 419-424.

Memmott, J., Waser, N. M., & Price, M. V. (2004). Tolerance of pollination networks to species extinctions. Proceedings of the Royal Society of London. Series B: Biological Sciences, 271(1557), 2605-2611.

Newman, M.E.J. (2010). Networks: An Introduction. Oxford University Press.

Pascual, M., & Dunne, J.A. (2006). Ecological Networks: Linking Structure to Dynamics in Food Webs. Oxford University Press.

Simmons, B. I., Cirtwill, A. R., Baker, N. J., Wauchope, H. S., Dicks, L. V., Stouffer, D. B., & Sutherland, W. J. (2019). Motifs in bipartite ecological networks: uncovering indirect interactions. Oikos, 128(2), 154-170.

Simmons, B. I., Beckerman, A. P., Hansen, K., Maruyama, P. K., Televantos, C., Vizentin‐Bugoni, J., & Dalsgaard, B. (2021). Niche and neutral processes leave distinct structural imprints on indirect interactions in mutualistic networks. Functional Ecology, 35(3), 753-763.
